# A Persistent Tuberculosis Outbreak in the UK Is Characterized by Hydrophobic *fadB4*-Deficient *Mycobacterium tuberculosis* That Replicates Rapidly in Macrophages

**DOI:** 10.1128/mbio.02656-22

**Published:** 2022-11-14

**Authors:** Robeena Farzand, Richard D. Haigh, Philip Monk, Pranabashis Haldar, Hemu Patel, Manish Pareek, Raman Verma, Michael R. Barer, Gerrit Woltmann, Lauren Ahyow, Heena Jagatia, Jonathan Decker, Galina V. Mukamolova, Andrea M. Cooper, Natalie J. Garton, Helen M. O’Hare

**Affiliations:** a Leicester TB Research Group, Department of Respiratory Sciences, University of Leicester, Leicester, UK; b Public Health England, Department of Health and Social Care in England, Government Agency, East Midlands, UK; c University Hospitals Leicester NHS Trust, University of Leicester, Leicester, UK; d National TB Unit, UK Health Security Agency, Government Agency, London, UK; Washington University School of Medicine in St. Louis

**Keywords:** *Mycobacterium tuberculosis*, genomes, lung infection, molecular epidemiology, tuberculosis

## Abstract

The genetic diversity of Mycobacterium tuberculosis can influence disease severity and transmissibility. To better understand how this diversity influences individuals and communities, we phenotyped M. tuberculosis that was causing a persistent outbreak in the East Midlands, United Kingdom. Compared to nonoutbreak isolates, bacilli had higher lipid contents and more hydrophobic cell surfaces. In macrophage infection models, the bacteria increased more rapidly, provoked the enhanced accumulation of macrophage lipid droplets and enhanced the secretion of IL-1β. Natural deletions in *fadB4*, *nrdB*, and *plcC* distinguished the outbreak isolates from other lineage 3 isolates in the region. *fadB4* is annotated with a putative role in cell envelope biosynthesis, so the loss of this gene has the potential to alter the interactions of bacteria with immune cells. Reintroduction of *fadB4* to the outbreak strain led to a phenotype that more closely resembled those of nonoutbreak strains. The improved understanding of the microbiological characteristics and the corresponding genetic polymorphisms that associate with outbreaks have the potential to inform tuberculosis control.

## INTRODUCTION

The genetic diversity of Mycobacterium tuberculosis and the genetic diversity of humans can influence disease severity and can potentially influence transmissibility, in part via strain-specific variation in the elicited immune response (1). Diversity between the nine lineages of M. tuberculosis, as well as diversity within lineages, is thought to influence host-pathogen interactions. Lineages 2 and 4, which are globally widespread (2), are associated with reduced or delayed proinflammatory immune responses, compared to those of other lineages (3, 4). Lineage 3, which is widespread in the regions of the world with the highest tuberculosis (TB) burden (5), has also been associated with reduced proinflammatory responses (6). The bacterial factors that favor transmission are less clear, and studies have found little association between lineage and transmission, and the interactions between pathogen and host genomics are also thought to be relevant (7–11).

TB is statutorily notifiable in the United Kingdom (UK). The incidence of TB is low (8.4 cases per 100,000 population in 2019), with the majority of cases occurring in patients born outside the UK (12). Under health protection regulations, isolates of M. tuberculosis have to be submitted to the national reference laboratories, which undertake typing.

More than half of the genome sequences of M. tuberculosis isolated in the UK are unique (not associated with another person with TB in the UK) (12), which suggests that the disease is caused by the reactivation of remotely acquired latent infections, usually from the country of origin. Among cases that are part of UK clusters, outbreaks that extend beyond the household and recognized close contacts contribute disproportionately to public health resources for TB control. Within this context, we have compared two contrasting outbreaks of drug-sensitive, lineage 3-associated TB recognized in the Midlands and have characterized the bacterial isolates associated with these outbreaks in order to understand the strain-specific characteristics that may be important determinants of epidemiological outcome.

The first outbreak (CH isolate) affected pupils and members of the staff at an urban secondary school (students 11 to 16 years of age) in 2000 (13, 14). The outbreak was characterized by extensive transmission and a high rate of rapid progression to active disease. Following an extensive public health screening exercise, 77 cases (70 students, 2 members of the staff, and 5 household contacts) of active TB and a further 257 cases (251 students and 6 household contacts) of latent TB infection (LTBI) were identified and treated. The CH isolate was characterized for gene deletions caused by IS6110 insertions and deletions (15) and was compared with other sequenced strains in a macrophage infection model (6). The outbreak was rapidly terminated by the implemented public health interventions, and no further cases related to the CH isolate appear to have arisen since.

In contrast, the second outbreak (Lro strain), recorded as cluster 7 in 2013 (16), was first identified in 2002 and continues to cause cases of incident TB in the region, most recently in 2021 (12). The majority of the cases associated with this outbreak have been in people with one or more social risk factors and have been clinically characterized by a paucity of clinical symptoms but extensive cavitatory pulmonary disease. Early cases treated with a supervised regimen of first line antituberculous medication for 6 months had a high rate of relapse, leading to a regional decision for all such cases to receive an extended 9-month course of treatment.

Key differences between this outbreak and the earlier school outbreak are the socially diverse networks and the challenges of identifying exposed contacts to test and treat. Repeated exercises of extended public health screening have been conducted with the emergence of new cases, and to date, 61 cases of active TB have been identified and treated. Contrary to other TB cases in the Midlands, routine contact screening methods have been insufficient to halt the outbreak. While the challenges associated with the complex social networks have contributed to the persistence of this outbreak, the specific clinical phenotypes of infection suggest that strain-related features are important, particularly with regard to more effective and extensive transmission that is driven by delayed diagnosis.

The importance of strain-related factors is further supported by our recent study that demonstrated the differences in the immunological profiles of patients in the Lro cluster. Comparing the blood transcriptomes of patients with active TB and the contacts of tuberculosis patients (17), the signatures from the Lro M. tuberculosis subjects differed qualitatively from those of the other subjects in three aspects: a more rapid progression of the signature in the period before diagnosis, a slower/smaller response of the signature during treatment, and, for the patients with active TB, a relative underexpression of the immune modules that are typically high.

Therefore, we hypothesized that bacterial factors could contribute to the virulence and transmission of the persistent outbreak (Lro). Among the putative bacterial features, the cell surface properties were of interest, as the specialization of the hydrophobic, immune modulatory cell surface is thought to have evolved during the emergence of M. tuberculosis as a human pathogen (18). Also, the variation in surface-exposed lipids and proteins has been associated with strain-specific differences in phagocytosis and in the induction of immune response and virulence, including hypervirulence traits (19).

To investigate our hypothesis, we compared the genetic nature, the microbiological characteristics, and the immunological impact of Lro outbreak isolates with isolates from the earlier contained CH outbreak as well as with isolates of M. tuberculosis lineages 2 and 4.

## RESULTS

### Identification of the outbreak.

The genetic link between the early Lro outbreak cases was established by MIRU-VNTR typing, and this cluster was one of the validating clusters in the current whole-genome sequencing (WGS) approach (16).

### Genetic characterization of Lro *M. tuberculosis*.

**FIG 1:**
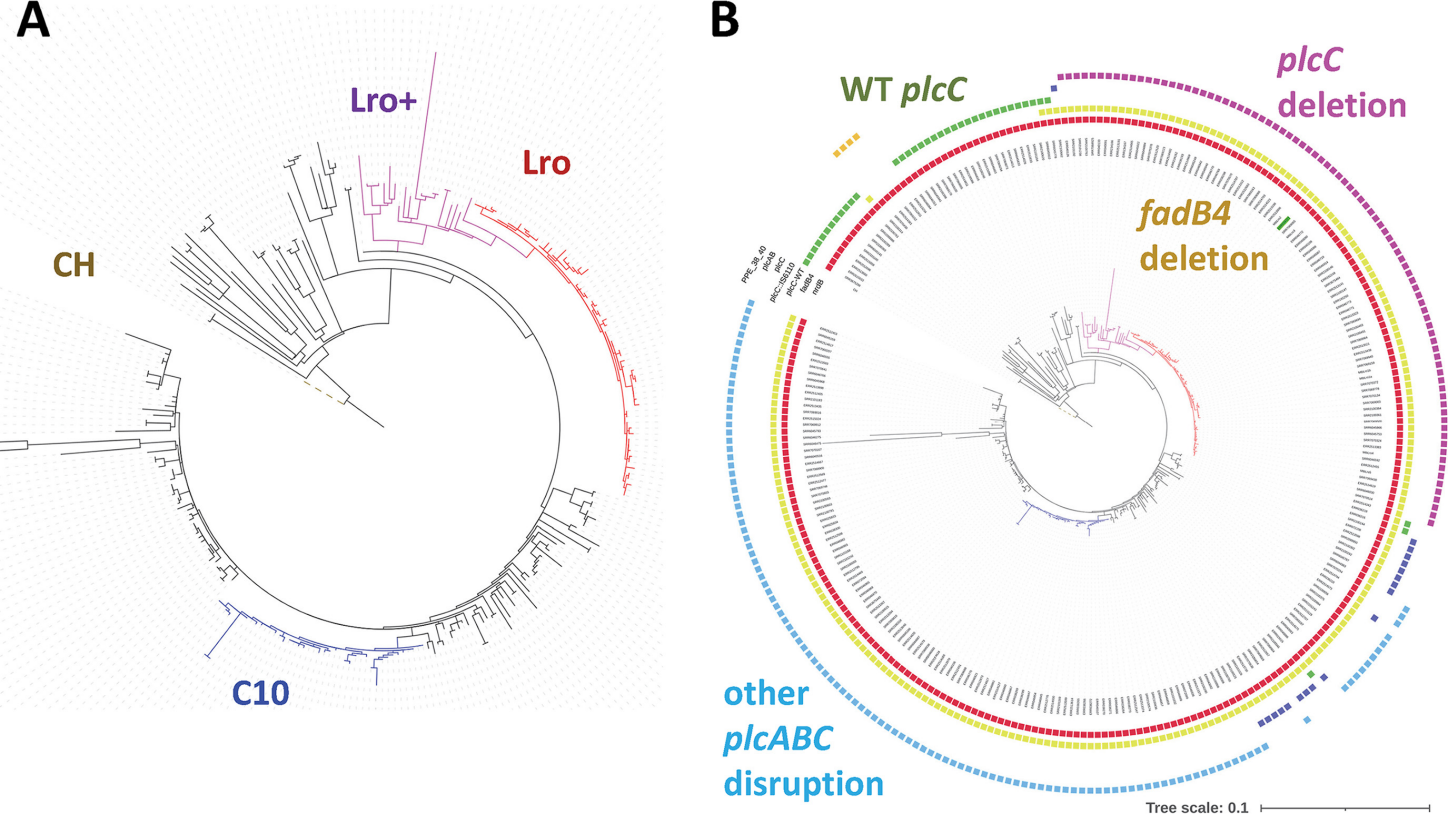
Phylogenetic analysis of the genomes of M. tuberculosis lineage 3 isolated in the Midlands. (A) The Lro outbreak is a distinct cluster of 63 genomes that differ by an average of <10 SNPs (shown in red) and is part of a larger clade, which is labeled as Lro+ (purple, includes 6 genomes from isolates from Africa, whereas all other genomes are isolates from the Midlands). Genomes related to Lro were selected by the presence of the *nrdB* deletion, and the tree was rooted using the genome of the CH outbreak strain (dashed brown line). A second cluster has been labeled C10 (blue). (B) The Lro outbreak is characterized by 3 deletions that are unique to the Lro+ clade: *nrdB*, *fadB4*, and *plcC*. The presence, absence, or disruption of genes in each genome is depicted with colored boxes around the edge of the tree. Red, deletion in *nrdB*; green, intact *plcC*; yellow, disrupted *fadB4*. Purple, dark blue, pale blue, and orange indicate polymorphisms in or near the *plcC* cluster, a deletion in *plcC* or *plcAB*, an insertion, and a PPE disruption, respectively. The genome highlighted in green represents the isolate studied in Fig. 2–4.

To understand the genetic characteristics of the M. tuberculosis isolates associated with the persistent Lro outbreak, isolates were sequenced and compared to the geographically and temporally close school CH outbreak, which was controlled and resulted in less severe disease profiles (14, 15). We found that the isolates from the persistent outbreak are distinct from those of the school outbreak and of the other regional lineage 3 isolates due to a unique combination of genomic deletions (*nrdB*, *fadB4*, and *plcC*) and cluster-specific single-nucleotide polymorphisms (SNPs). To identify the closest comparator isolates sequenced in the region, a process was developed (see Materials and Methods) to identify lineage 3 genomes (2,464 out of 7,282 genomes) with the *nrdB* deletion as an identifier (261 out of 2,299 lineage 3 genomes were filtered for data quality). A phylogenetic analysis of the isolates from the Lro cluster with its regional comparators (Fig. 1) indicated a mix of migration-related (unique) and local (clustered) transmission. Importantly, the isolates from the persistent Lro outbreak form a cluster of 63 highly related genomes, with an average of fewer than 10 SNPs between the members of the cluster (Fig. 1). There are 15 defining SNPs that are unique to the Lro clade and are present in all of the Lro clade (Table 1).

**TABLE 1:** Defining the SNPs of the Lro cluster

Position	Gene	Synonym	Reference > Lro	Coding change	Gene function
314778	*rv0262c*	*aac*	77A > C	Q26P	Aminoglycoside resistance
361159	*rv0296c-rv0297*	*rv0296c-PE_PGRS5*	Intergenic T > C	Intergenic	
627340	*rv0536*	*galE3*	93C > T	Synonymous	Epimerase (cell wall)
1199991	*rv1075c*	*rv1075c*	380G > A	G127D	Conserved exported
1519740	*rv1353c*	*rv1353c*	246C > G	I82M	Probably transcriptional regulator
1728055	*rv1527c*	*pks5*	355G > A	G119S	Polyketide synthase
1814105	*rv1614*	*lgt*	935C > T	A312V	Maturation of lipoproteins
1986839	*rv1754c-rv1755c*	*rv1754c-plcD*	Intergenic T > C	Intergenic	
2011808	*rv1777*	*cyp144*	1153T > G	C385G	Cytochrome P450 heme monooxygenase
2363071	*rv2101*	*helZ*	2832C > A	D944E	Helicase
3183542	*rv2872*	*vapC43*	161G > A	R54H	Toxin antitoxin system
3265893	*rv2934*	*ppsD*	3646G > A	A1216T	PDIM biosynthesis
3630522	*rv3250c*	*rubB*	45G > C	E15D	Rubredoxin
3646060	*rv3265c*	*wbbL1*	825G > A	Synonymous	Glycosyltransferase (cell wall)
4316405	*rv3843c*	*rv3843c*	192C > T	Synonymous	Membrane protein

Each of the *fadB4*, *plcC*, and *nrdB* deletions is rare, globally (present in 1, 0, and 9 out of the 286 M. tuberculosis completed genomes in GenBank). Using the PolyTB server to query 1,470 sequenced isolates from 18 locations (20), the 849-bp deletion in *fadB4* is a polymorphism that is present in a subset of lineage 3 isolates from Malawi, Tanzania, and Uganda, as well as the UK.

The presumed order of deletions is *nrdB* (in a redundant gene for nucleotide biosynthesis) followed by *fadB4* (encoding a putative oxidoreductase) followed by *plcC* (encoding a phospholipase and a known site for polymorphisms [21]). The disruption of the *plcABC*, *plcD* locus appears to have independently occurred three times following the loss of *fadB4* (Fig. 1). Thus, although the Lro outbreak is characterized by three deletions, other lineage 3 isolates in the Midlands have the same three genes disrupted, albeit with an independent disruption in the *plcABC* locus and with different SNPs. This includes 40 genomes that have most likely resulted from the continued transmission of an initial cluster reported in 2013 (cluster 10 in [16] and C10 in Fig. 1).

Our analysis shows that the persistent outbreak is associated with highly related M. tuberculosis isolates that are distinct from the CH school outbreak and from other locally obtained lineage 3 isolates (>10 SNPs from genomes from outside the cluster) (Fig. 1). The epidemiological profile also differs from that of the CH school outbreak in that it contains both single and clustered transmission events as well as ongoing transmission. The cluster-specific gene deletions and SNPs could contribute to the distinctive characteristics of this outbreak. The *fadB4* is of particular interest due to its presence in isolates from several continents.

### Microbiological characterization of Lro *M. tuberculosis*.

**FIG 2:**
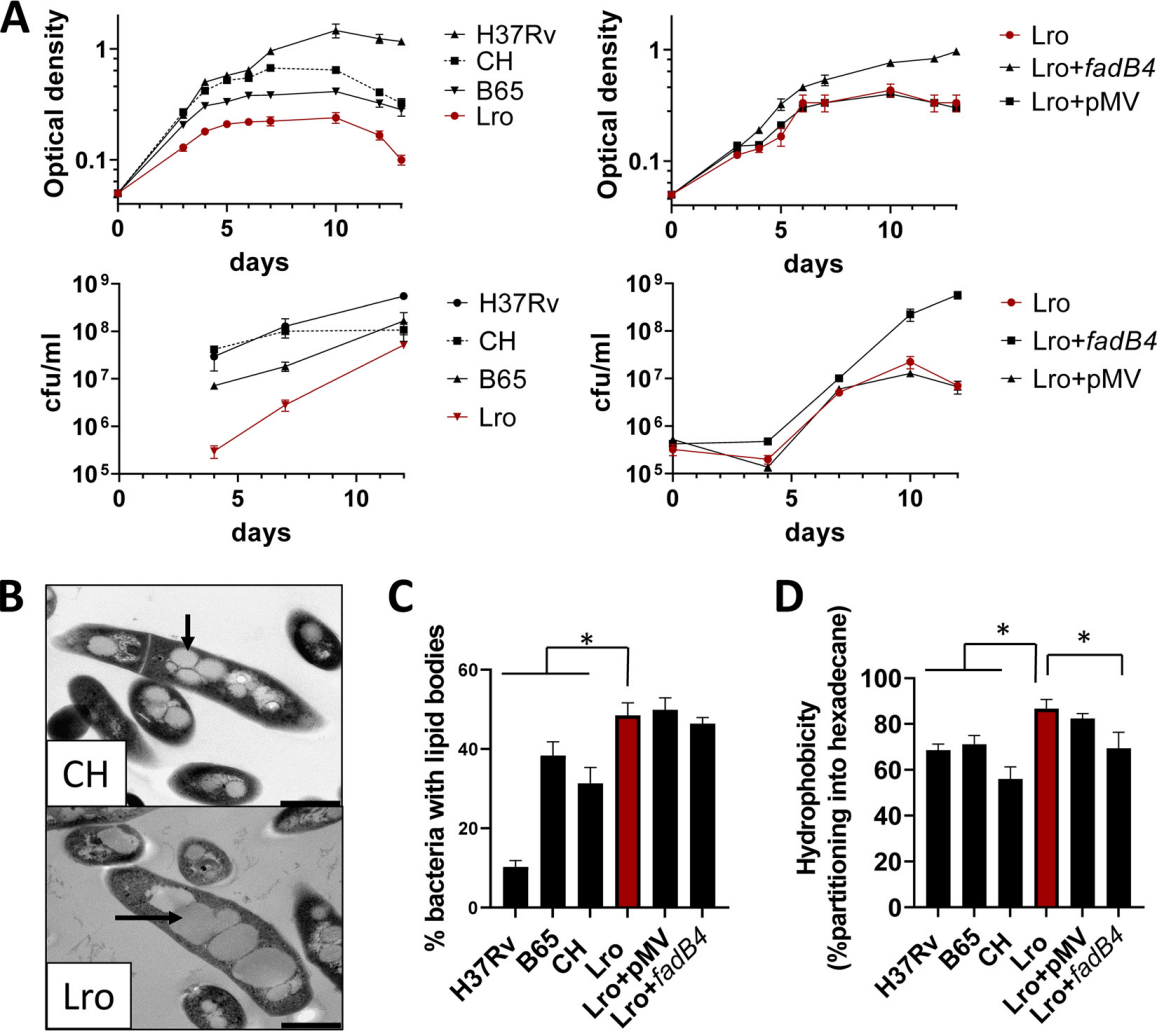
Phenotypic characterization of M. tuberculosis from the Lro outbreak and the restoration of the *fadB4* gene. (A) The Lro isolate was compared with the school outbreak strain (CH), type strain (H37Rv), and lineage 2 strain (B65) for growth in liquid culture. The optical density and CFU/mL for Lro were significantly lower (*P* < 0.05) than those of the other strains at day 7 (data are means and standard deviations of 3 to 4 replicates). Lro with the *fadB4* plasmid (Lro+*fadB4*) had significantly higher optical density and CFU/mL values at day 7 (*P* < 0.05). (B) Lro and CH bacilli each contained lipid bodies, indicated by arrows on the electron micrographs (scale bar of 500 nm). (C) Lro contained significantly more lipid bodies than CH, H37Rv, or B65: and the percentage of bacteria containing one or more lipid bodies was determined via Nile-red staining and fluorescence microscopy (mean and standard deviation of 300 cells for each strain; an asterisk indicates *P* < 0.05). (D) Lro, but not Lro+*fadB4*, was significantly more hydrophobic than the other strains (*P* < 0.05), as measured by the optical density change when bacteria moved into the hexadecane layer (mean and standard deviation of 3 experiments). The figure shows the microbiological profile of Lro M. tuberculosis that was isolated from one patient and then cultured in a common Mycobacterial growth medium (Sauton’s broth). Data were also gathered using the same isolate cultured in another common growth medium (Middlebrook 7H9) and using three other isolates from the Lro cluster (Fig. S1–S3).

Due to the genetic relatedness and the unique nature of the persistent outbreak (Lro), we wanted to investigate the microbiological features of the Lro strains. To do this, we compared an isolate (Lro) to a locally occurring lineage 3 isolate from the school outbreak (CH) as well as the well-characterized sequenced strain H37Rv (lineage 4) and the lineage 2 (Beijing) clinical isolate B65. As we also hypothesized that the *fadB4* contributed to the unique features of the outbreak, we also included in the comparison an Lro isolate transformed with an expression plasmid “*fadB4*” or an empty plasmid “pMV”. We chose two common M. tuberculosis culture media: Sauton’s broth, which has been used for studies of M. tuberculosis lipids, and Middlebrook 7H9, which has been used more generally (22, 23). The results using Sauton’s broth are reported in Fig. 2, and the parallel experiments using Middlebrook 7H9 are reported in the supplemental material.

The Lro isolate grew more slowly in the exponential phase (*P* < 0.05) and reached a lower maximum density (*P* < 0.05), and the cultures of Lro showed clumping during the stationary-phase (visible clumping coincided with a decline in optical density and greater variation between replicates) (Fig. 2A). In contrast, the addition of the *fadB4* gene resulted in enhanced and prolonged growth, relative to the original Lro isolate or the control plasmid (*P* < 0.05) (Fig. 2A, right panel). In parallel experiments in the more complex Middlebrook 7H9 broth, which generally supports faster growth of M. tuberculosis, the Lro isolate grew similarly to the lineage 2 isolate but more slowly than the other comparators, and the addition of the *fadB4* gene modestly enhanced the growth of the Lro isolate (Fig. S1).

10.1128/mbio.02656-22.1 FIG S1 Phenotypic characterization of M. tuberculosis from the Lro outbreak and the restoration of the *fadB4* gene: growth rate. (A) Lro was compared with the school outbreak strain (CH), type strain (H37Rv), and lineage 2 strain (B65) for growth in liquid culture in Middlebrook 7H9 broth with ADC and Tween 80, 0.05%. The optical density and CFU/mL values for Lro were significantly lower (*P* < 0.05) than those of H37Rv or CH at day 7 (data are means and standard deviations of 3 to 4 replicates). (B) Lro with the *fadB4* plasmid (Lro+*fadB4*) had significantly higher optical density and CFU/mL values at day 7 (*P* < 0.05). (C and D) The colony forming units per mL of the cultures shown in (A) and (B) were measured by serial dilution onto Middlebrook 7H10 agar with ADC. Panel (E) shows the growth rate for the first 4 days of the M. tuberculosis cultures from (A) and (B). Panel (F) shows the growth rate during the first 4 days for the M. tuberculosis cultures from Fig. 1, which used a simpler broth: Sauton’s medium. Download FIG S1, TIF file, 2.6 MB. Copyright © 2022 Farzand et al. 2022 Farzand et al. https://creativecommons.org/licenses/by/4.0/ This content is distributed under the terms of the Creative Commons Attribution 4.0 International license.

The presence of lipid bodies in M. tuberculosis is a marker of bacterial physiology (23) and, for clinical samples, of treatment outcome (24, 25). To investigate the tendency of the Lro isolate to develop lipid bodies, we compared bacterial strains by their cell structure, as observed via electron microscopy, and by their abilities to bind a lipophilic fluorescent stain. We found that the lineage 3 isolates Lro and CH developed abundant lipid bodies (Fig. 2B; Fig. S2) and that the proportion of bacilli with lipid bodies was significantly higher for Lro (*P* < 0.05) (Fig. 2B; Fig. S3). Lro transformed with the *fadB4* plasmid had a reduced proportion of cells with lipid bodies, but this result did not reach statistical significance (Fig. 2C; Fig. S3), suggesting that other genetic differences between the isolates may account for differences in lipid accumulation.

Given the putative involvement of *fadB4* in cell envelope biosynthesis and the role of the cell envelope in both virulence and bacterial clumping, Lro was next characterized for surface hydrophobicity via hexadecane partitioning. We found that the Lro isolate was significantly more hydrophobic than were the comparator strains and that the addition of the *fadB4* gene to the Lro isolate significantly reduced hydrophobicity (Fig. 2D; Fig. S3).

Our data support the hypothesis that the Lro isolate has a significantly different growth fitness in defined media, has an increased capacity to generate lipid bodies, and has an increased hydrophobicity, relative to comparator strains. Our data also support the claim that these characteristics can be reversed by restoring the *fadB* gene into the isolate.

### Interaction of Lro *M. tuberculosis* with macrophages.

**FIG 3:**
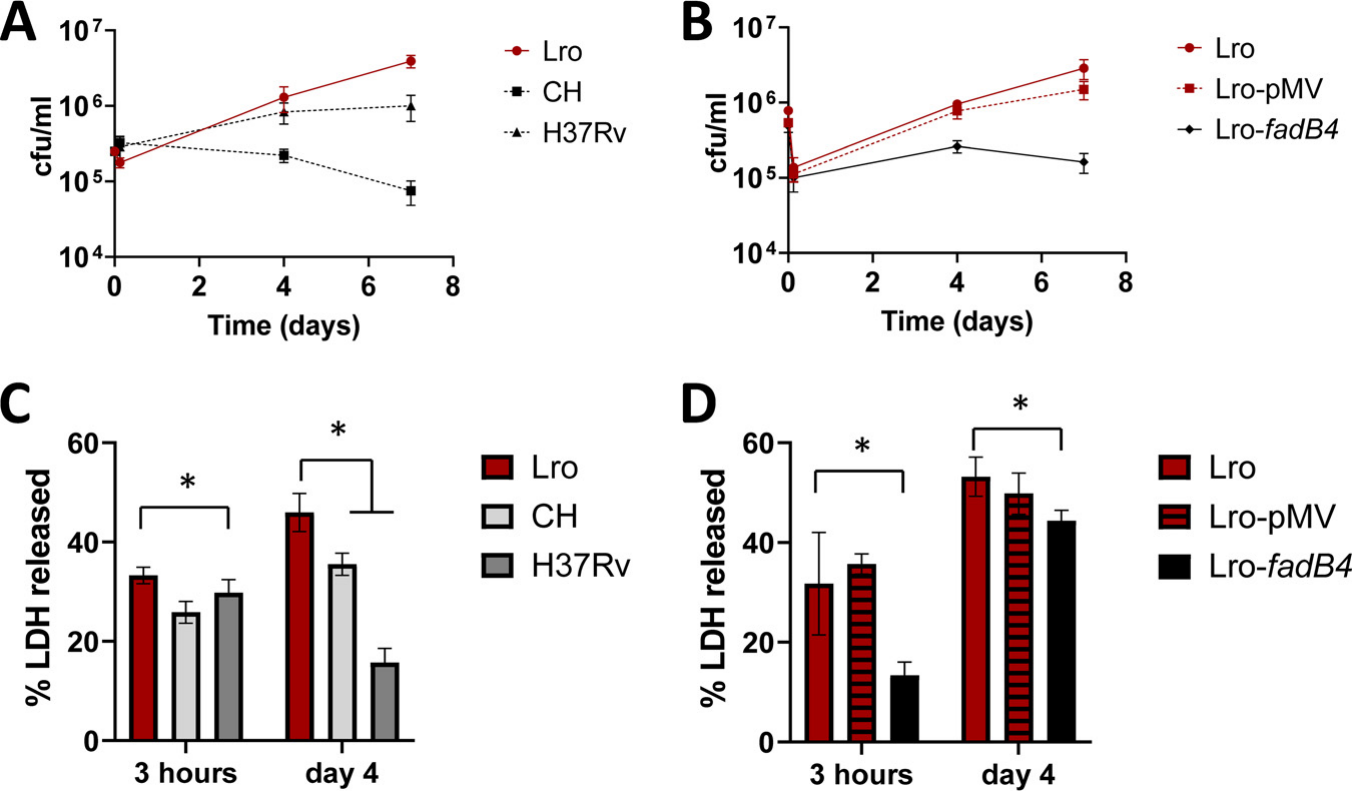
Lro increased more rapidly than did the comparator strains in THP-1 monocytes and caused greater cell damage. The THP-1 were primed with 10 ng/mL INF-γ and were then infected with the indicated strains of M. tuberculosis at a 1:1 ratio. (A and B) Intracellular bacteria were recovered via the lysis of THP-1 and quantitated by CFU. Data are the means and standard deviations of at least four infections. At 7 days, the CFU values for Lro were significantly higher than those for CH, H37Rv, or Lro-*fadB4* (*P* < 0.05). (C and D) Cytotoxicity was determined via the measurement of lactate dehydrogenase activity in the cell culture medium of infected cells and was compared to the LDH activity when 100% of the cells were lysed using detergent. 100% represents the LDH activity when THP-1 were fully lysed using detergent. Data are the means and standard deviations of at least four wells. An asterisk indicates *P* < 0.05.

The bacterial cell surface may have an impact on a range of virulence-associated activities, and the early interaction with the host innate immune cells is particularly important in defining the immunological response to infection (26). We hypothesized that the Lro isolate might differ from the contained school outbreak isolate (CH) or nonoutbreak strains in how it interacts with innate cells. To test this hypothesis, we studied the responses of macrophages to the Lro and CH isolates and chose the lineage 4 comparator (H37Rv) instead of the other nonoutbreak strain (B65), as the type strain H37Rv is the most studied. To compare Lro with other M. tuberculosis in terms of its ability to infect and replicate in human cells, we needed a cell line for high reproducibility. We chose the THP-1 human monocyte cell line, which is widely used in tuberculosis research, as virulence phenotypes in this model (higher cytotoxicity and a faster increase in intracellular bacilli) have been previously been associated with higher numbers of bacilli in patient sputum before treatment (27). Using PMA-treated THP-1, we compared infection dynamics over time. We found that the uptake of the isolates was similar but that the size of the Lro bacterial population within the macrophages increased over 7 days to a greater degree than did that of the H37Rv or the CH isolate (Fig. 3A). We also found that the presence of the *fadB4* gene reduced the size of the bacterial population over 7 days, compared to the untransformed or empty plasmid isolate (Fig. 3B). Increases in bacterial population were likely due to intracellular bacilli, as M. tuberculosis replicates little in cell culture medium (RPMI) (28).

Preliminary microscopic analysis indicated that the Lro-infected macrophages accumulated lipids in their cytoplasm within hours of infection, whereas little or no lipid staining was seen in the macrophages infected with the comparator isolates of M. tuberculosis (Fig. S4). Therefore, we measured the release of LDH into the media to measure the impact of the bacteria on macrophage viability. We found that the Lro isolates induced more LDH release than did the comparators, and this was reduced in the presence of the *fadB4* gene (Fig. 3C and D).

10.1128/mbio.02656-22.2 FIG S2 Microscopic characterization of M. tuberculosis from the Lro outbreak. The M. tuberculosis Lro isolate was compared with an isolate from the school outbreak (CH) and a lineage 2 isolate (B65) by growth in Middlebrook 7H9 and subsequent electron microscopy. Lro bacilli contained lipid bodies, indicated by arrows on the electron micrographs (scale bar of 500 nm). The quantitation of lipid bodies is presented in Fig. 2C. Download FIG S2, TIF file, 2.5 MB. Copyright © 2022 Farzand et al. 2022 Farzand et al. https://creativecommons.org/licenses/by/4.0/ This content is distributed under the terms of the Creative Commons Attribution 4.0 International license.

10.1128/mbio.02656-22.3 FIG S3 Phenotypic characterization of M. tuberculosis from the Lro outbreak: additional isolates. The Lro isolate that was sequenced and characterized most thoroughly in this study is designated “Lro”. Three additional isolates from the Lro outbreak, designated Lro2, Lro3, and Lro4, were characterized in terms of growth rate, lipid body content, and hydrophobicity, exactly as was done in Fig. 1, giving similar results to those observed with Lro (no significant difference in growth, lipid staining, or hydrophobicity). (A) Growth in Sauton’s broth was measured by optical density. (B) The percentage of bacteria containing lipid bodies was determined by Nile-red staining cells grown as in panel A and then performing fluorescence microscopy on 300 cells for each isolate. (C) Hydrophobicity was measured by the optical density change in the aqueous phase when the bacteria partitioned into a hexadecane layer (mean and standard deviation of 3 experiments). Download FIG S3, TIF file, 0.6 MB. Copyright © 2022 Farzand et al. 2022 Farzand et al. https://creativecommons.org/licenses/by/4.0/ This content is distributed under the terms of the Creative Commons Attribution 4.0 International license.

10.1128/mbio.02656-22.4 FIG S4 Lipid accumulation during the early responses of THP-1 to infection with the M. tuberculosis Lro isolate and the comparator isolates (CH and H37Rv). The THP-1 were infected with the indicated isolate of M. tuberculosis at a MOI of 1:1. After 3 h, the cells were fixed and stained with Auramine and Nile-red and were analyzed via fluorescence microscopy. Circles show Auramine-stained, intracellular bacilli. Arrows show areas of lipid accumulation. Download FIG S4, TIF file, 2.2 MB. Copyright © 2022 Farzand et al. 2022 Farzand et al. https://creativecommons.org/licenses/by/4.0/ This content is distributed under the terms of the Creative Commons Attribution 4.0 International license.

These data support the hypothesis that the Lro isolate has improved intracellular fitness, relative to the comparator isolates. The data also support an association between this improved fitness and the loss of the *fadB4* gene.

**FIG 4:**
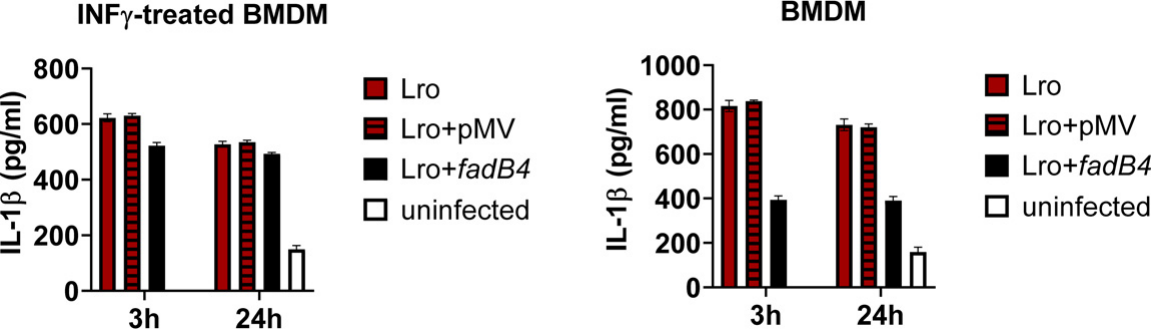
Influence of *fadB4* deletion on cytokine release by Lro-infected BMDM. Lro provoked a higher IL-1β release from bone marrow-derived macrophages than did Lro+*fadB4*, as measured by ELISA, whether or not the macrophages had been primed with INF-γ (10 ng/mL). Additional conditions and the TNF-α release are provided as supplementary figures. BMDM were infected at a MOI of 1:1. Supernatants were collected after 3 and 24 h for the ELISA. Data are the means and standard deviations of 4 replicates, using BMDM from 2 mice. An asterisk indicates *P* < 0.05.

The initial interaction with innate cells, such as macrophages, results in the release of cytokines that can alter the behavior of the macrophages and of the rest of the immune system. We hypothesized that the gene deletions drive alterations to the interactions between the bacteria and the macrophages and that this impacts cytokine production. To test this hypothesis, we needed primary cells, and, in order to have sufficient cells with sufficient reproducibility, we used murine bone marrow-derived macrophages (BMDM). We infected BMDM with Lro and CH isolates and H37Rv, and we measured the secretion of proinflammatory cytokines IL-1β and TNF-α. Prior to infection, the macrophages were either untreated or were primed with INF-γ, which can suppress the production of active IL-1β by macrophages (29, 30). Initial experiments showed that TNF-α secretion did not differ between the strains (Fig. S5), whereas IL-1β secretion was higher for Lro than for CH under all conditions tested (Fig. S6). To determine whether this difference in the induction of the critical inflammatory cytokine IL-1β was associated with *fadB4* gene expression, we compared the response of the bone marrow-derived macrophages to the transformed Lro isolate. We found that the Lro isolate (both untreated and with the empty plasmid) evoked significantly higher IL-1β secretion than did the Lro isolate transformed with the *fadB4* plasmid, and the difference was greatest early after infection and in the absence of prestimulation with INF-γ (Fig. 4). Lipid staining and microscopy of the infected BMDM revealed that infection with the Lro isolate, compared to the CH isolate, led to qualitatively higher lipid staining of the BMDM (Fig. S7), similar to the experiments with THP-1 (Fig. S4).

10.1128/mbio.02656-22.5 FIG S5 Comparing the TNF-α release from BMDM infected by different isolates of M. tuberculosis. BMDM (with or without a pretreatment of 10 ng/mL INF-γ) were infected with the indicated M. tuberculosis isolate at a MOI of 1:1. Supernatants were collected after 3 and 24 h for ELISA. Data are the means and standard deviations of 4 replicates, using BMDM from 2 mice. The TNF-α response was not significantly different between the different isolates (*P* > 0.05). Download FIG S5, TIF file, 0.7 MB. Copyright © 2022 Farzand et al. 2022 Farzand et al. https://creativecommons.org/licenses/by/4.0/ This content is distributed under the terms of the Creative Commons Attribution 4.0 International license.

10.1128/mbio.02656-22.6 FIG S6 Comparing IL-1β release from BMDM infected by either Lro, CH or H37Rv M. tuberculosis. BMDM (with or without pretreatment with 10 ng/mL INF-γ) were infected with the indicated M. tuberculosis isolate at MOI of 1:1. Supernatants were collected after 3 and 24 h for ELISA. Data are the mean of 2 replicates, using BMDM from 2 mice. The IL-1β response was higher for Lro infection than for CH infection under each condition tested, and the mean IL-1β response to Lro was significantly higher than that to CH at 24 h (*P* < 0.05). Download FIG S6, TIF file, 0.9 MB. Copyright © 2022 Farzand et al. 2022 Farzand et al. https://creativecommons.org/licenses/by/4.0/ This content is distributed under the terms of the Creative Commons Attribution 4.0 International license.

10.1128/mbio.02656-22.7 FIG S7 Lipid accumulation during the early responses of BMDM to infection with the M. tuberculosis Lro isolate and the comparator isolates (CH and H37Rv). INF-γ-treated BMDM were infected with the indicated isolate of M. tuberculosis at a MOI of 1:1. After 3 h, cells were fixed and stained with Auramine and Nile-red and were analyzed via fluorescence microscopy. Circles show Auramine-stained, intracellular bacilli. Arrows show areas of lipid accumulation. Download FIG S7, TIF file, 2.2 MB. Copyright © 2022 Farzand et al. 2022 Farzand et al. https://creativecommons.org/licenses/by/4.0/ This content is distributed under the terms of the Creative Commons Attribution 4.0 International license.

## DISCUSSION

The M. tuberculosis responsible for the persistent Lro outbreak is genetically and phenotypically distinct from the previously characterized 2000 Leicester school outbreak strain (10, 11) and is also distinct from the other comparator isolates. These differences translate to distinctive outcomes in infection models: Lro M. tuberculosis increased more rapidly in a macrophage infection model and caused macrophages to accumulate lipid droplets and secrete more proinflammatory IL-1β. This phenotype is likely to influence the course of the Lro outbreak at the patient and community levels and is relevant for understanding the impact of genetic diversity in TB more widely.

The outbreak strain contains a deletion in *fadB4* that is relatively rare in clinical isolates. The putative function of *fadB4* is the oxidation of fatty acids or polyketides that are cell envelope components, and the gene is induced in macrophages (31). The knockout of *fadB4* in M. tuberculosis H37Rv led to hypervirulence in animal and cell-based infection models (32).

Some of the phenotypic characteristics of Lro are likely to be associated with the natural deletion of *fadB4*, as Lro+*fadB4* more closely resembled the school outbreak strain CH. These phenotypes were consistent with changes in lipid biosynthesis: more lipid bodies in the bacilli and greater surface hydrophobicity. Changes in the lipid content would influence the fate of the bacilli in infection models by three routes: surface lipids influence interactions with phagocytic and nonphagocytic cells, certain lipids are virulence determinants, and bacterial and host lipids are an energy store (33). Consistent with this view, some of the strain-specific characteristics of Lro in macrophage infections were reduced or abrogated by the reintroduction of *fadB4*: cytotoxicity, an increase in intracellular bacilli, and the IL-1β response.

Comparing Lro, which has natural *fadB4* deletion, with the *fadB4* knockout studied previously (32), the strains are alike in the rapid increase in intracellular bacteria, but they differ in the measured TNF-α response, which may be due to the different infection model (the previous work used a RAW cell line and did not measure IL-1β).

Strain specific reduction in the IL-1β response has recently been linked to more severe disease (34). However, the classification of the disease severity of patients in the Lro cluster is complex, as even patients with extensive pathology exhibited relatively moderate symptoms. Perhaps surprisingly, given the high IL-1β induction by Lro in BMDM, patients in the Lro cluster had a muted blood transcriptome signature compared to the immune modules that are generally high in active TB (17). This could be a potential reason why patients reported fewer symptoms and were diagnosed later. However, the size of the study was limited by the size of the outbreak, and statistical analysis was not possible.

INF-γ is a mediator of activation with an essential role in the restriction of the growth of intracellular bacteria and a role in the induction of lipid droplets in macrophages (35). Although INF-γ has proinflammatory features, it can suppress the generation of proinflammatory active IL-1β by macrophages (30). Lro differed significantly from other strains in two macrophage infection models in the presence and absence of priming by INF-γ. Notably, Lro induced lipid droplet formation in macrophages, a hallmark of TB, which is also seen in animal models and in various cell infection models. Infection-induced changes in macrophage fatty acid metabolism have been viewed as beneficial to the pathogen in that they supply fatty acids or are a part of a protective proinflammatory response (33). The factors that trigger lipid accumulation in TB are thought to include metabolic reprogramming, immune signaling, and the inhibition of autophagy, with the latter also inducing IL-1β secretion (33, 36).

Strain specific variation in lipid droplet induction has recently been studied in the context of high and low transmission M. tuberculosis (based on whether household contacts became infected). Low-transmission M. tuberculosis accumulated more rapidly in a murine aveolar macrophage cell-line and induced more lipid droplets (LD) and more IL-1β release (11). Mouse model data were also available, in which the low transmission strain induced more diffuse pathology and fewer caseating lesions (which might break down and lead to transmission). There are some similarities between Lro and the low transmission high IL-1β, high LD phenotype, albeit in a different cell type. However, the presence of cavitatory lesions in patients affected by Lro resembles the caseating granuloma response to high transmission Mtb in the mouse model.

Previous reports of strain-specific phenotypes relating to outbreaks present a complex picture, with outbreaks being characterized as having high or low proinflammatory phenotypes (6, 19, 37). The apparent discrepancies may arise from the different bacterial and human factors (with differing mechanisms) underpinning the different outbreaks.

It is interesting to note that the three gene losses in the Lro outbreak are shared (with some conserved SNPs) by a larger clade of isolates in the UK and in Africa (named “Lro+” in Fig. 1). A comparison of disease severity and transmission features in these communities could improve knowledge on the human factors contributing to outbreaks. Similarly, the three gene losses in the Lro outbreak are shared, without conserved SNPs, by the cluster 10 clade in the UK (C10 in Fig. 1). A comparison of the clinical features of cluster 10 with Lro could illuminate the relative contribution of gene deletions versus SNPs in the evolution of the outbreak.

Studies of outbreaks are influenced by national and regional differences in health, living conditions, BCG vaccination, and HIV status, in addition to the various choices of methodologies for bacterial and infection work. Furthermore, clinical isolates of M. tuberculosis easily accumulate mutations that increase the growth rate on laboratory media, which can include the loss of virulence-associated lipids. Here, we noted that the strain-specific differences in the growth rate were more pronounced in Sauton’s broth than in the Middlebrook medium and that the relatively slow growth of Lro isolates in medium contrasted with their relatively rapid accumulation in infected macrophages. Standardized conditions and criteria with which to characterize M. tuberculosis isolates would accelerate progress in understanding the mechanisms by which bacterial genetic variation influences disease and transmission.

In conclusion, we report a *fadB4* deficient, lipid-rich bacillus that accumulates rapidly in macrophages, strongly induces lipid droplets and IL-1β secretion, and has been transmitted efficiently in a population in which TB prevalence and HIV infection rates are low. The divergence in disease pathology is likely the underlying cause of differences in the infectiousness of the source case. However, since the current findings are based on a small sample size, larger confirmatory studies are required to draw broader inferences that individual strains of M. tuberculosis induce specific disease features.

## MATERIALS AND METHODS

### Bacterial strains and culture.

M. tuberculosis strains: H37Rv (ATCC 25618), CH was isolated during the school outbreak in 2001 (14, 15); (the Beijing lineage strain B-65 was isolated in Cape Town [38]); Lro was isolated during the current outbreak (2017 to 2019; Public Health England reference AA503-3) on Lowenstein-Jensen slant and was scraped directly into 7H9 with 25% (wt/vol) glycerol for storage at −80°C. a subculture of Lro was minimized to 2 passages to preserve the phenotype and genotype of the isolate. Lro+*fadB4* and Lro+pMV were constructed from Lro via transformation with pMV306 or its derivative and were cultured in the presence of 25 μg/mL kanamycin.

Three additional Lro isolates (Lro2, Lro3, and Lro4) were measured in parallel with Lro and gave similar results in terms of growth rates and hydrophobicity (see the data in the supplemental material).

M. tuberculosis was cultured in Sauton’s medium and in Middlebrook 7H9 medium for phenotypic characterization and in Middlebrook 7H9 to prepare for cell infection. Sauton’s medium contained, per 1 L, 4 g asparagine, 2 g citric acid, 0.5 g K_2_HPO_4_, 0.5 g MgSO_4_7 H_2_O, and 0.05 g ferric ammonium citrate, supplemented with 1% (wt/vol) glycerol and 0.05% (wt/vol) Tween 80. Middlebrook 7H9 (BD Difco) medium was supplemented with 10% (vol/vol) albumin-dextrose supplement (ADC: 2.5 g of BSA fraction V, 420 mg NaCl, 1 g dextrose, and 2 mg catalase in 50 mL) plus 0.05% (wt/vol) Tween 80. Cultures were incubated in conical flasks with filter caps (Fisher Scientific) at 37°C with shaking. Where required, 7H10 (BD Difco) agar was used, supplemented with 10% (vol/vol) oleic acid-ADC, which is ADC with 5.5% (vol/vol) oleic acid (1% [wt/vol] solution in 0.2 M NaOH).

Growth curves were initiated using the exponential-phase culture as the inoculum, normalized by the optical density. Growth curves presented on the same axes were gathered contemporaneously, using the same batch of broth.

### Genomics and phylogenetic tree analysis.

This research used the Alice High Performance Computing Facility at the University of Leicester.

Genomic DNA from four isolates (Lro, Lro2, Lro3, Lro4) was used for whole-genome sequencing by MicrobesNG (Birmingham), using the Nextera XT libraries on an Illumina platform. Paired-end sequences (250 bp) were assembled to contigs using SPAdes 3.9.0, and the genome assembly statistics were determined using QUAST 4.3. Genomes and raw reads were deposited in GenBank under accession number PRJNA882339.

7,282 sets of Illumina paired-end sequencing data, identified as having been derived from Mtb strains in the Birmingham PHE culture collection (see Table 2 for literature sources and accession numbers), were downloaded from the European Nucleotide Archive. Downloaded sequences were then screened (by k-mer matching, the assembly of lineage defining regions, and BLASTn analysis) to identify strains carrying the lineage 3 defining deletions RD750 and *ctpG*. 2,464 strains were identified. The FastQ sequences for all of the potential L3 strains were assembled to genomes as sets of contigs (using SPAdes [39]), and the non-Mtb sequences were filtered out (using k-mer matching with bbmap (40)). Only those strains with filtered genome sizes >4.2 Mbp but <4.5 Mbp were considered for further analysis (*n* = 2,299).

**TABLE 2:** Genome sequences

Study accession	Related publication(s)	Total bham samples	L3 bham samples	Good L3 bham samples
PRJEB2221	16	494	155	142
PRJEB3373	16	2	2	2
PRJNA282721	16	1262	524	420
PRJNA401515	55	932	248	241
PRJNA302362	56, 57	1,874	583	578
PRJEB25991	58	2,718	952	916
Total		7,282		2,299

The assembled Mtb genomes were aligned to the H37Rv genome sequence (NC_000962.3) using NUCmer (41), and SNPs were called using the MUMmer script show-snps (41). The SNPs were filtered with vcftools (42), removing those in Mtb repeat regions (using PHE’s repetitive region SNP filter; T. Walker personal communication), and were then made into multi-fasta files (http://code.google.com/p/vcf-tab-to-fasta) for phylogeny analysis with FastTree (43). Genome sequences were examined by BLASTn (44) for RD750 and *ctpG* deletions and also for the novel deletions (i.e., *nrdB*, *fadB4*, *Rv1645c*, and *plcC*) that have been observed in the Lro_strain_10698/10699 genomes. The phylogeny mapping and BLASTn results indicated that the *nrdB* deletion would be a useful identifier for a manageably sized group of L3 WGSs that mapped close to the Loughborough cluster. These strains were selected for further in-depth study (*n* = 261 ENA-derived strains, plus the sequencing done in this study).

The read sequences (FastQ format) from 264 *nrdB* deletion-carrying Midlands Mtb were aligned to the H37Rv genome (NC_000962.3) using the program bwa_mem (45), and the SNPs/indels were called using Samtools (46) and VarScan (47) (minimum depth of 10 reads and a variant fraction of 0.8). Low confidence SNPs/indels were then filtered out using both the PHE repetitive region filter (T. Walker, personal communication) and our own filter that was created with an in-house script that identifies regions of <10 base depth coverage in any of the *nrdB* deletion WGSs. Only the filtered SNPs were then used to create multi-fasta files (Bergey CM, 2012; vcf-tab-to-fasta; http://code.google.com/p/vcf-tab-to-fasta) for tree building using FastTree (43) and, more recently, IQ-TREE (48, 49), and they were then visualized in iTOL (50).

### Genetic modification of Lro.

The *fadB4* gene (696 bp) was amplified from H37Rv genomic DNA by PCR. Overlapping PCR was used to add the *hsp60* promoter sequence. The sequence-verified insert was subcloned into EcoRI-HindIII-digested pMV306 to create pMV-*fadB4*. pMV306 is an integrative plasmid in which the expression cassette of pMV361 is replaced by a multiple cloning site (51). The primers used are listed in Table 3. Plasmids pMV306 and pMV+*fadB4* were transformed into the Lro strain by electroporation to create Lro+pMV and Lro+*fadB4*.

**TABLE 3:** Primer sequences

Primer ID	Sequence (5′–3′)	Features
GroEl2_EcoRI_F	CGGAATTCATGGGCCGAACATACTCACC	EcoRI restriction digestion site (underlined)
GroEl2_R	TGCGAAGTGATTCCTCCGG	
fadB4_F	CCGGAGGAATCACTTCGCAATGCGCGCGGTACGGGT	Overlapping sequence of GroEl2_R (underlined)
fadB4_HindIII_R	TTAGTCGCGCACGCGTAGTAAGCTTGGG	HindIII restriction digestion site (underlined)
pmv306_F	CACAGGAGTTGCAACCCG	For pMV306 screening
pmv306_R	CACTTCCGGGTGTACTCC	

### Measurement of intracellular lipid bodies via fluorescence microscopy.

The bacteria were grown until the OD_580_ reached 0.5, at which time they were applied to glass slides, air dried, heated to 100°C for 30 s, and exposed to formaldehyde vapour for 24 h. Staining by Auramine and then by Nile-red was performed as described (22). Fluorescence microscopy was conducted using an inverted microscope (Nikon Eclipse Ti) with a mercury halide light source. The images were recorded with a DS-Qi1Mc digital monochrome cooled camera, using NIS Elements (Nikon Instruments Ltd.). For Auramine imaging an A 31015: Auramine (excitation of 460 ± 25 nm; emission of 550 ± 25 nm; Chroma Technology) filter set was used, and for Nile-red, a 49008 (excitation of 560 ± 20 nm; emission of 630 ± 37.5 nm, Chroma Technology) filter set was used for epifluorescence microscopy. Automated image analysis was used to score the bacterial cells on the basis of the presence or absence of lipids, using an R script as described (52).

### Measurement of bacterial surface hydrophobicity by hexadecane-aqueous buffer partitioning.

Bacteria were grown until the OD_580_ reached 0.8 to 0.9 and were then heat killed (85°C for 2 h). The killed cells were washed twice in phosphate-urea-magnesium buffer (22.2 g K_2_HPO_4_ 3H_2_O, 7.26 g KH_2_PO_4_, 1.8 g urea, 0.2 g of MgSO_4_ 7H_2_O per 1L, pH 7.1) and were then normalized to an OD_580_ of 0.5. Cell surface hydrophobicity was assayed by the hexadecane partitioning assay as described (18). The hydrophobicity data presented are the proportions of cells removed from the aqueous phase (as measured by OD_580_) after partitioning with hexadecane. Results are expressed as the mean ± the standard deviation (SD) of at least three independent experiments.

### Analysis of bacteria via electron microscopy.

Bacteria were grown in the indicated broth (Sauton’s or Middlebrook) to an OD_580_ of 0.6 to 0.8 and were washed with PBS before fixation in 2.5% (vol/vol) glutaraldehyde (Agar Scientific, UK) in PBS for 24 h. The fixative was removed via two washes in PBS. The samples were embedded, stained, and dehydrated as described (53). The presented images are representative of a minimum of 20 images (>100 bacteria) taken for each strain and condition.

### Preparation of *M. tuberculosis* stocks for macrophage infection.

M. tuberculosis strains were cultured to the log phase (OD_580_ of 0.5 to 0.7) in 10 mL of 7H9. This starter culture was used to inoculate 200 mL 7H9. The cells were harvested by centrifugation when the OD_580_ value reached 0.8, washed with 10% glycerol in PBS, and then resuspended in RPMI. The OD_580_ was adjusted to approximately 0.8 to 0.9, and aliquots were stored at −80°C. A single aliquot was defrosted, and the CFU counts were determined for each strain for the multiplicity of infection (MOI) calculation.

### Infection of THP-1 cells and assay of cytotoxicity and intracellular bacteria.

M. tuberculosis stocks were prepared for macrophage infection as described in the supplementary methods. The human monocytic cell line THP-1 (ATCC TIB TIB-202) was maintained in RPMI 1640 supplemented with 10% (vol/vol) FBS, 2 mM l-glutamine, and 1 mM sodium pyruvate at 37°C and 5% CO_2_.

THP-1 cells were differentiated at a cell density of approximately 2× 10^5^ cells/mL using 100 ng/mL phorbol myristate acetate (PMA) for 24 h. Then, the medium was replaced, and the cells were incubated for a further 24 h. The cells were pretreated with recombinant IFN-γ (Sigma-Aldrich) at 10 ng/mL for 24 h before infection. Macrophages (approximately 2× 10^5^ cells/mL) were infected with M. tuberculosis at a MOI of 1 and were incubated for 3 h at 37°C and 5% CO_2_. After 3 h, the macrophages were washed 3 times with prewarmed PBS and were then treated with amikacin (200 μg/mL) in supplemented RPMI for 1 h. Amikacin was removed via three washes with PBS, and then the cells were incubated in supplemented RPMI at 37°C and 5% CO_2_ for 7 days.

Cytotoxicity was determined by an assay of lactate dehydrogenase (LDH) activity in the supernatant of infected cells, using a commercial cytotoxicity detection kit (Sigma-Aldrich). The supernatant was filtered twice through a 0.2 μm filter to remove risk of infection, and then the kit was used, according to the manufacturer’s instructions. In parallel, the cells were fully lysed using Triton X-100 (0.1% [vol/vol]) to determine the LDH activity that resulted from 100% lysis.

The intracellular bacteria were quantified via the lysis of infected THP-1 cells, using 0.2% (vol/vol) Triton X-100 at room temperature for 10 min, serial dilution, and the counting of CFU. Three biological replicates were conducted at each time point.

### Infection of BMDM, assay of cytotoxicity, and intracellular bacteria and cytokine release.

Murine bone marrow-derived macrophages were isolated as described (54) and infected with M. tuberculosis at a MOI of 1:1 or were pretreated with INF-γ at 10 ng/mL for 24 h before infection. Supernatant collected after 3 and 24 h was filtered to remove infection risk and was then assayed for LDH as described above and for TNF-α and IL1γ by enzyme-linked immunosorbent assay (ELISA), following the manufacturer’s instructions (BioLegend).

Where the microscopy of infected THP-1 or BMDM was required, the cells were fixed by exposure to formaldehyde vapour for 24 h. The staining by Auramine and Nile-red and the microscopy were performed as described for staining the bacterial cells. Infected cells were identified by the presence of Auramine-stained bacilli, and the presented images are representative of each strain and condition.

### Statistical analysis.

The following data sets did not show a detectable difference from normality (Shapiro-Wilk test [*P* > 0.05] and a visual inspection of Q-Q-plots): bacterial growth, hydrophobicity, LDH release, lipid staining, and cytokine release. Results were expressed as the mean ± the SD from at least three independent experiments. Unpaired *t* tests were used to evaluate differences between the strains, with the exception of the results of lipid body counting, which were tested using a one-way analysis of variance (ANOVA). The data were presented using the GraphPad Prism software package.
