# Supplementary figures and images for: A Persistent Tuberculosis Outbreak in the UK Is Characterized by Hydrophobic fadB4-Deficient Mycobacterium tuberculosis That Replicates Rapidly in Macrophages
Source: mBio. 2022 Nov 14;13(6):e02656-22. doi: 10.1128/mbio.02656-22 (PMC9765663; doi:10.1128/mbio.02656-22)

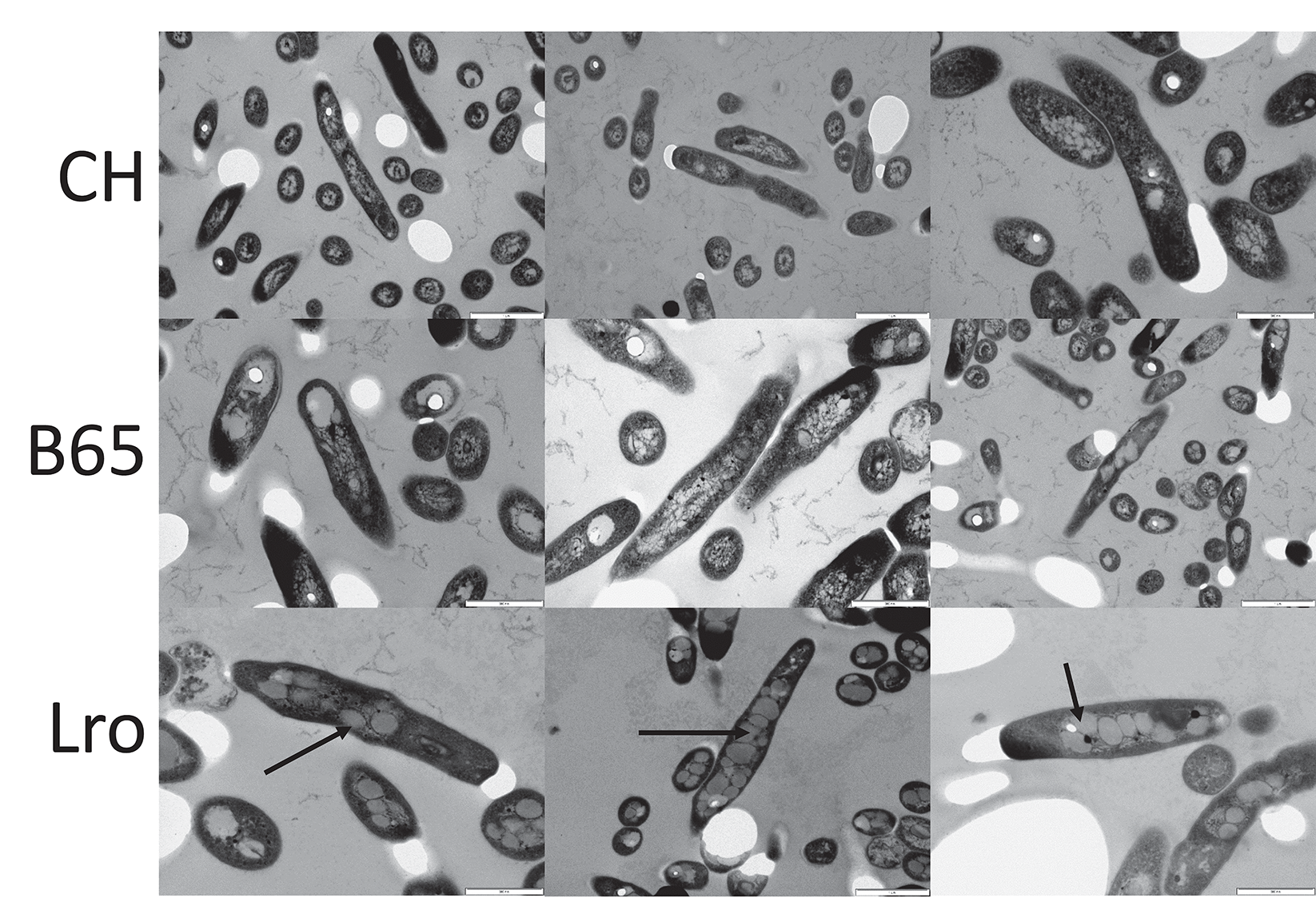

Supplement: FIG S2 [file mbio.02656-22-s0002.tif]

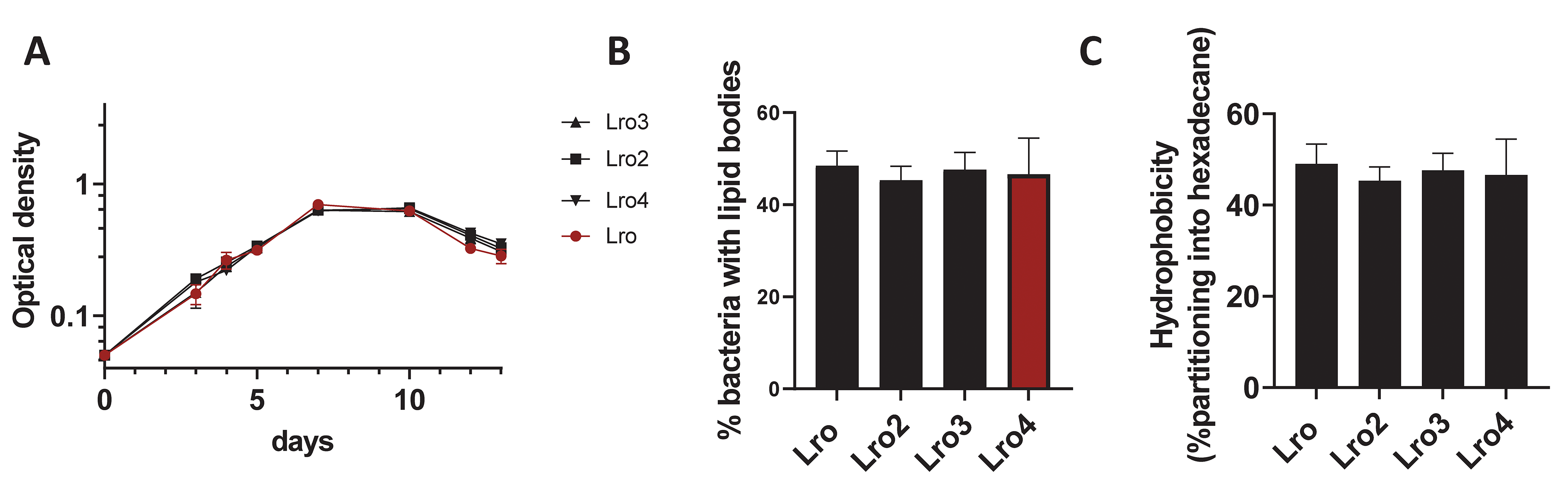

Supplement: FIG S3 [file mbio.02656-22-s0003.tif]

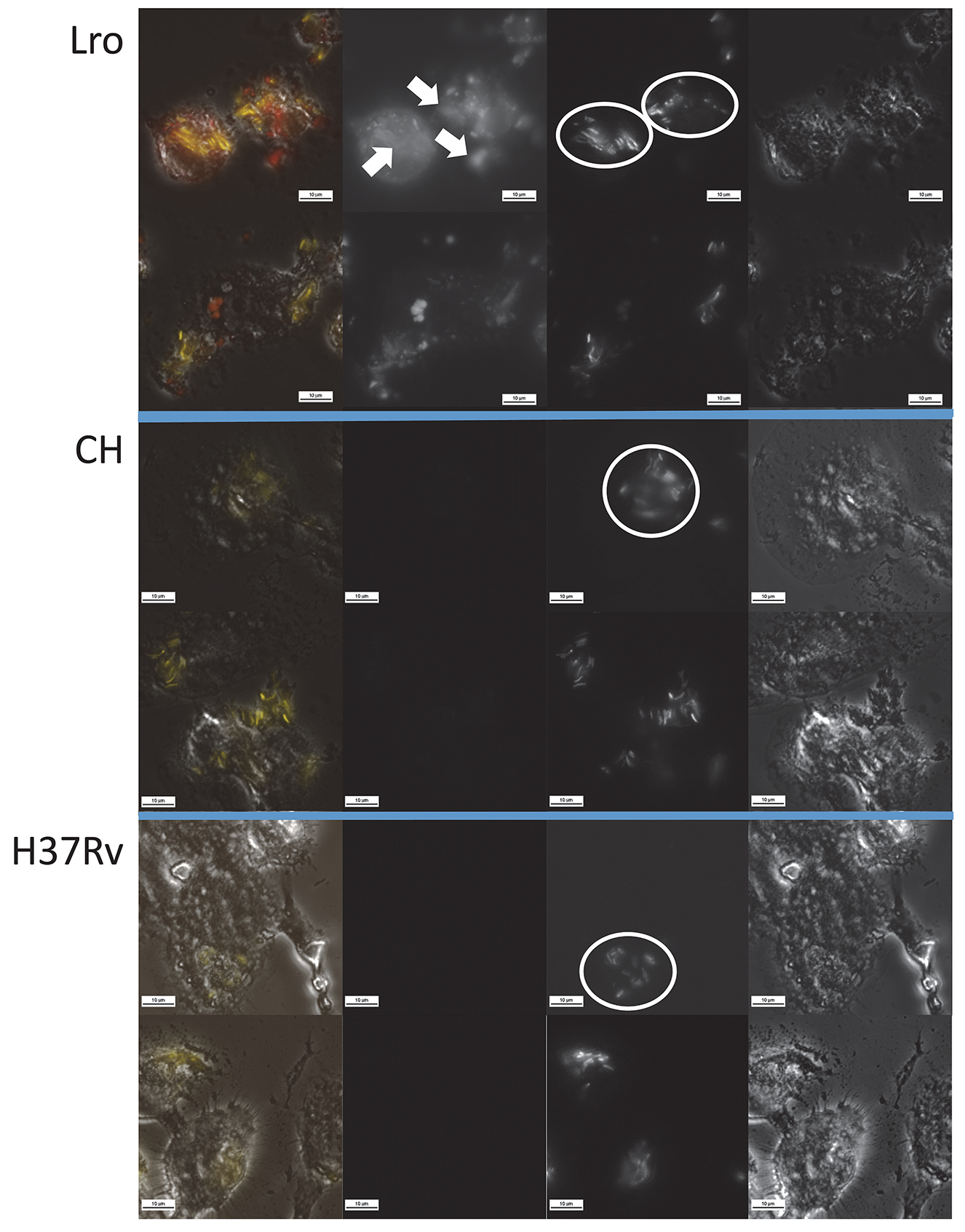

Supplement: FIG S4 [file mbio.02656-22-s0004.tif]

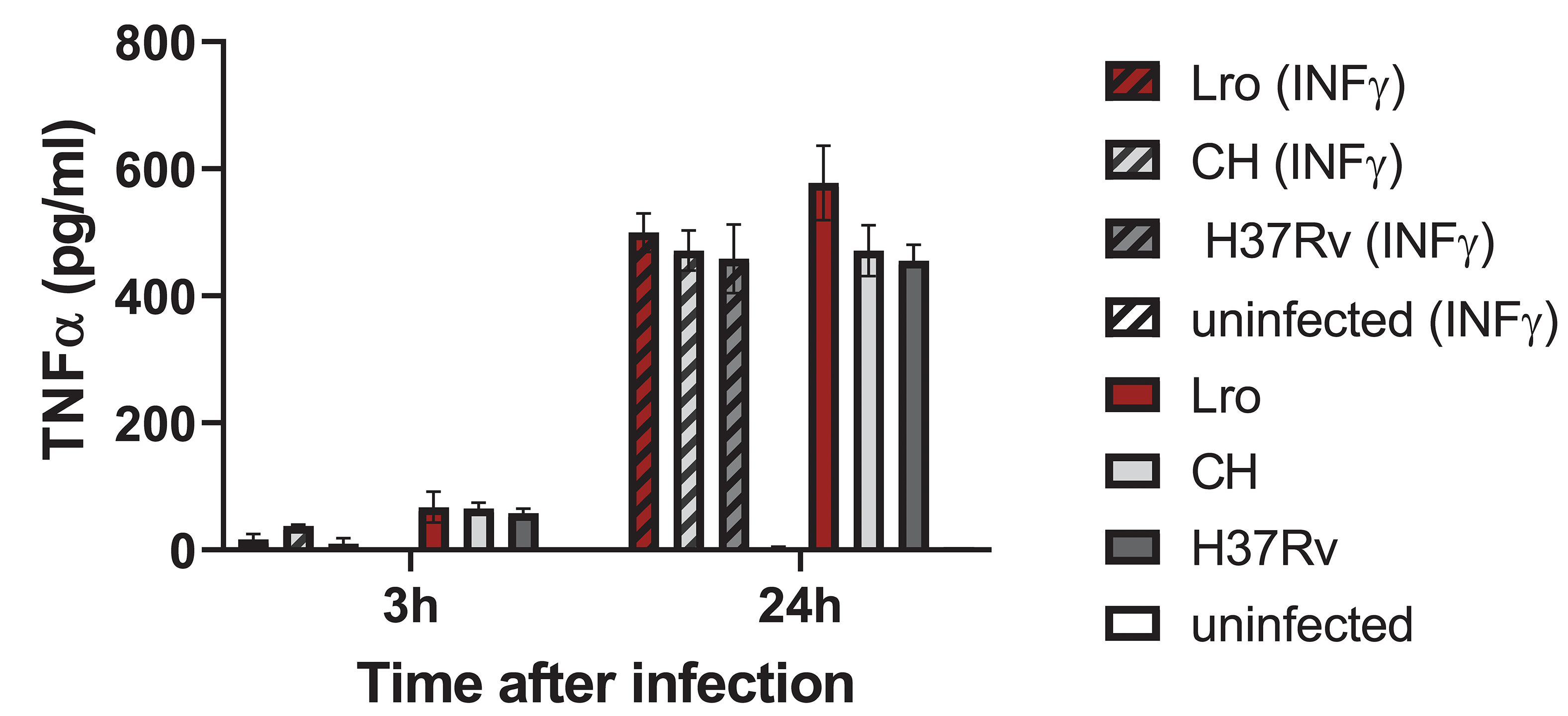

Supplement: FIG S5 [file mbio.02656-22-s0005.tif]

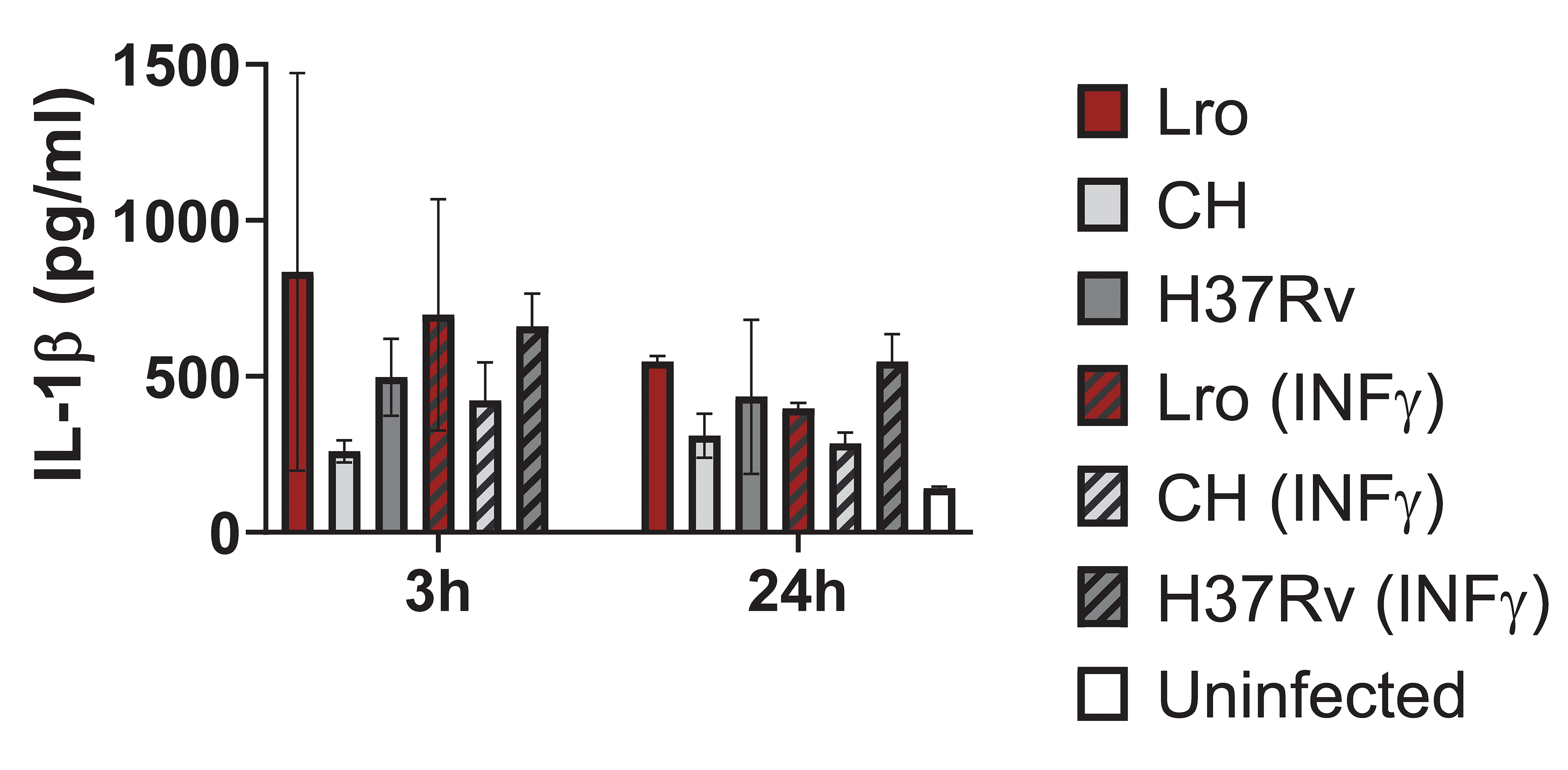

Supplement: FIG S6 [file mbio.02656-22-s0006.tif]

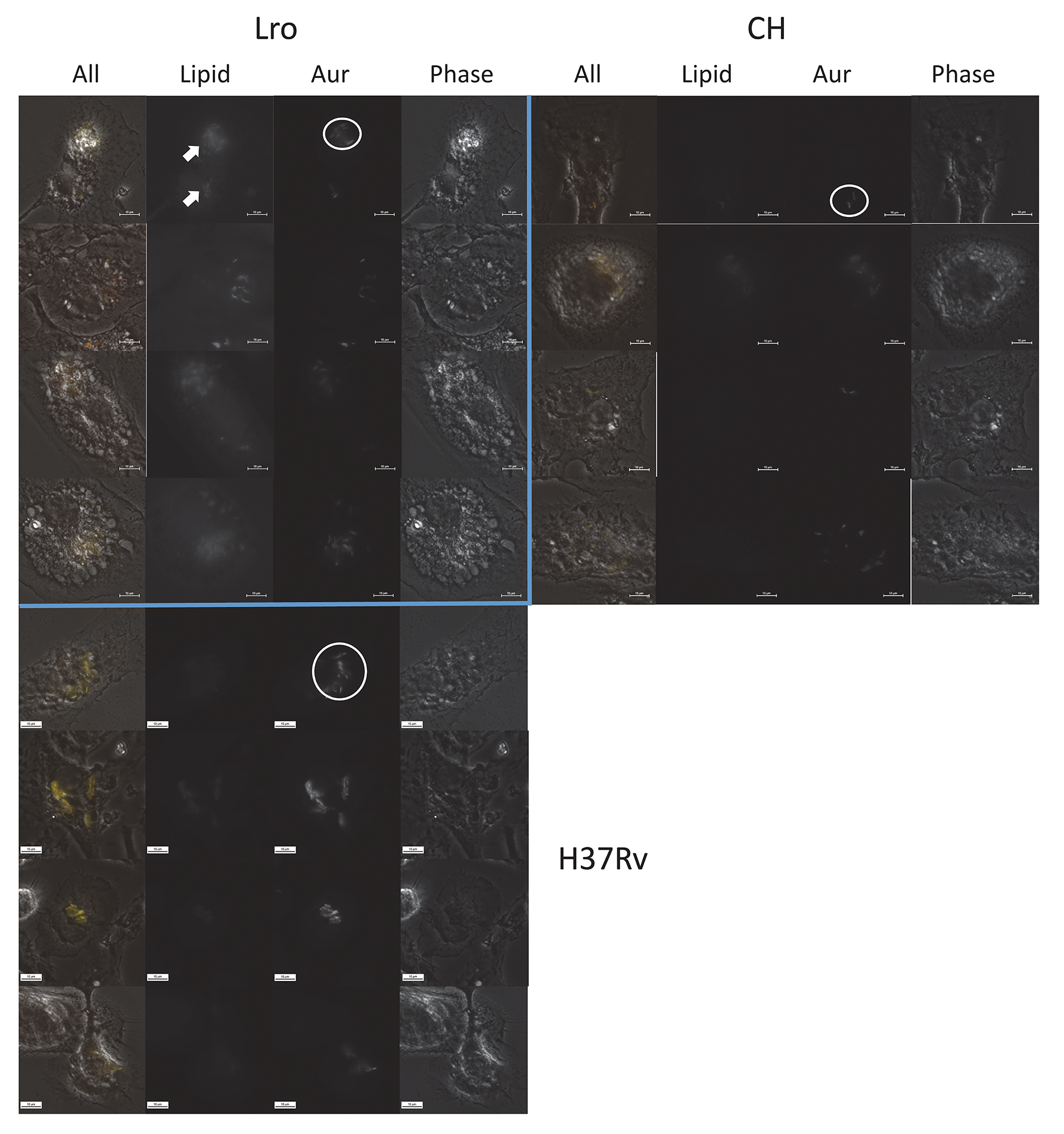

Supplement: FIG S7 [file mbio.02656-22-s0007.tif]
